# Concurrent Kleene Algebra with Observations: From Hypotheses to Completeness

**DOI:** 10.1007/978-3-030-45231-5_20

**Published:** 2020-04-17

**Authors:** Tobias Kappé, Paul Brunet, Alexandra Silva, Jana Wagemaker, Fabio Zanasi

**Affiliations:** 8 ENS/CNRS, Université Paris-Saclay, Cachan, France; 9 University of Duisburg-Essen, Duisburg, Germany; University College London, London, UK

## Abstract

Concurrent Kleene Algebra (CKA) extends basic Kleene algebra with a parallel composition operator, which enables reasoning about concurrent programs. However, CKA fundamentally misses *tests*, which are needed to model standard programming constructs such as conditionals and $\mathsf {while}$-loops. It turns out that integrating tests in CKA is subtle, due to their interaction with parallelism. In this paper we provide a solution in the form of Concurrent Kleene Algebra with Observations (CKAO). Our main contribution is a completeness theorem for CKAO. Our result resorts on a more general study of CKA “with hypotheses”, of which CKAO turns out to be an instance: this analysis is of independent interest, as it can be applied to extensions of CKA other than CKAO.

*Acknowledgments.* This work was partially supported by the ERC Starting Grant ProFoundNet, grant code 679127. We acknowledge support from the EPSRC grants EP/S028641/1 (A. Silva); EP/R020604/1 (F. Zanasi); EP/R006865/1 (P. Brunet).

## 1 Introduction

*Kleene algebra with tests* (KAT) is a (co)algebraic framework [17,19] that allows one to study properties of imperative programs with conditional branching, i.e. if-statements and while-loops. KAT is build on Kleene algebra (KA) [6,16], the algebra of regular languages. Both KA and KAT enjoy a rich meta-theory, which makes them a suitable foundation for reasoning about program verification. In particular, it is well-known that the equational theories of KA and KAT characterise rational languages [27,21,16] and guarded rational languages [17] respectively. Efficient procedures for deciding equivalence have been studied in recent years, also in view of recent applications to network verification [3,8,28].

Concurrency is a known source of bugs and hence challenges for verification. Hoare, Struth, and collaborators [11], have proposed an extension of KA, *Concurrent Kleene Algebra* (CKA), as an algebraic foundation for concurrent programming. CKA enriches the basic language of KA with a parallel composition operator $\cdot \parallel \cdot$. Analogously to KA, CKA also has a semantic characterisation for which the equational theory is complete, in terms of rational languages of *pomsets* (words with a partial order on letters) [23,24,15].

The development of CKA raises a natural question, namely how tests, which were essential in KAT for the study of sequential programs, can be integrated into CKA. At first glance, the obvious answer may appear to be to merge KAT with CKA, yielding Concurrent Kleene Algebra with Tests (CKAT) — as attempted in [12]. However, as it turns out, integrating tests into CKA is quite subtle and this naive combination does not adequately capture the behaviour of concurrent programs. In particular, using the CKAT framework of [12] one can prove that for any test $b$ and CKAT program $e$:

$$0 \quad \leq_{\mathsf{KAT}} \quad b \cdot e \cdot \overline{b} \quad \leq_{\mathsf{CKA}} \quad e \parallel (b \cdot \overline{b}) \quad \equiv_{\mathsf{KAT}} \quad e \parallel 0 \quad \equiv_{\mathsf{CKA}} \quad 0$$

thus $b \cdot e \cdot \overline{b} \equiv_{\mathsf{CKAT}} 0$, meaning no program $e$ can change the outcome of any test $b$. Or equivalently, and undesirably, that any test is an invariant of any program!

The core issue is the identification in KAT of sequential composition $\cdot$ and Boolean conjunction $\wedge$. In the concurrent setting this is not sound as the values of variables — and hence tests — can be changed between the two tests.

In order to fix this issue, we have presented *Kleene Algebra with Observations* (KAO) in previous work [13]. Algebraically, KAO differs from KAT in that conjunction of tests $b \wedge b'$ and their sequential composition $b \cdot b'$ are distinct operations. In particular, $b \wedge b'$ expresses a single test executed *atomically*, whereas $b \cdot b'$ describes two distinct executions, occurring one after the other. As mentioned above, this distinction is crucial when moving from the sequential setting of KA to the concurrent setting of CKA, as actions from another thread that happen to be scheduled after $b$ but before $b'$ may as well change the outcome of $b'$.

This newly developed extension of KA enables a novel attempt to enrich CKA with the ability to reason about programs that also have the traditional conditionals: in this paper, we present Concurrent Kleene Algebra with Observations (CKAO) and show that it overcomes the problems present in CKAT.

The traditional plan for developing a variant of (C)KA is to define a separate syntax, semantics, and set of axioms, before establishing a formal correspondence with the base syntax, semantics and axioms of (C)KA proper, and arguing that this correspondence allows one to conclude soundness and completeness of the axioms w.r.t. the semantics, as well as decidability of equivalence in the semantics. Instead of such a tailor-made proof, however, we take a more general approach by first proposing CKA with hypotheses (CKAH) as a formalism for studying extensions of CKA, akin to how Kleene algebra with hypotheses [5,18,20,7] can be used to extend Kleene algebra. We then apply CKAH to study CKAO, but the meta-theory developed can also be applied to extensions other than CKAO.

Using the CKAH formalism, we instantiate CKAO as CKAH with a particular set of hypotheses, and we immediately obtain a syntax and semantics; we can then use the meta-theory of CKAH to argue completeness and decidability in a modular proof, which composes results about CKA [15] and KAO [13].

The technical roadmap of the paper and its contributions are as follows.

- We introduce Concurrent Kleene Algebra with Hypotheses (CKAH), a formalism for studying extensions of CKA; this is a concurrent extension of Kleene Algebra with Hypotheses (Section 4). We show how CKAH is sound with respect to rational pomset languages closed under an operation arising from the set of hypotheses. We propose techniques to argue completeness of the extended set of axioms with respect to the sound model as well as decidability of equivalence, capturing methods commonly used in literature to argue completeness and decidability for extensions of (concurrent) KA.

- We prove that CKAO can be presented as an instance of CKAH, for a certain set of hypotheses (Section 5). This gives us a sound model of CKAO 'for free'. We then prove that the axioms of CKAO are also complete for this model, and that equivalence is decidable, using the techniques developed previously.

We conclude this introduction by giving an example of how hypotheses can be added to CKA to include the meaning of primitive actions. Suppose we were designing a DSL for recipes, specifically, the steps necessary, and their order. A recipe to prepare cookies might contain the actions $\mathsf{mix}$ (mixing the ingredients), $\mathsf{preheat}$ (pre-heating the oven), $\mathsf{chill}$ (chilling the dough) and $\mathsf{bake}$ (baking the cookies). Using these actions, a recipe like "mix the ingredients until combined; chill the dough while pre-heating the oven; bake cookies in the oven" may be encoded as $\mathsf{mix}^* \cdot (\mathsf{chill} \parallel \mathsf{preheat}) \cdot \mathsf{bake}$. Now, imagine that we have only one oven, meaning that we cannot bake two batches of cookies concurrently. We might encode this restriction on concurrent behaviour by forcing the equation

$$(e \cdot \mathsf{bake} \cdot f) \parallel (g \cdot \mathsf{bake} \cdot h) = (e \cdot \mathsf{bake} \parallel g) \cdot (f \parallel \mathsf{bake} \cdot h) + (e \parallel g \cdot \mathsf{bake}) \cdot (\mathsf{bake} \cdot f \parallel h)$$

As a consequence of this hypothesis, one could then derive properties such as

$$\mathsf{bake} \parallel (\mathsf{bake} \cdot \mathsf{mix}) = \mathsf{bake} \cdot \mathsf{bake} \cdot \mathsf{mix} + \mathsf{bake} \cdot \mathsf{mix} \cdot \mathsf{bake}$$

In a nutshell, this paper provides an algebraic framework — CKAH — together with techniques for soundness and completeness results. The framework is flexible in that different instantiations of the hypotheses generate very different algebraic systems. We provide one instantiation — CKAO — that enables analysis of programs with both concurrency primitives and Boolean assertions. This is the first sound and complete algebraic theory to reason about such programs.

For the sake of brevity, some proofs appear in the extended version [14].

## 2 Preliminaries

We recall basic definitions on pomset languages, used in the semantics of CKA, which generalise languages to allow letters in words to be partially ordered. We fix a (possibly infinite) alphabet $\Sigma$. When defining sets parametrised by $\Sigma$, say $\mathsf{S}(\Sigma)$, if $\Sigma$ is clear from the context we use $\mathsf{S}$ to refer to $\mathsf{S}(\Sigma)$.

**Posets and Pomsets** Pomsets [9,10] are labelled posets, up to isomorphism.

**Definition 2.1 (Labellet poset).** *A* labelled poset *over $\Sigma$ is a tuple $\mathbf{u} = \langle S, \leq, \lambda \rangle$, where $S$ is a finite set (the* carrier *of $\mathbf{u}$), $\leq_{\mathbf{u}}$ is a partial order on $S$ (the* order *of $\mathbf{u}$), and $\lambda : S \to \Sigma$ is a function (the* labelling *of $\mathbf{u}$).*

We will denote labelled posets by bold lower-case letters $\mathbf{u}$, $\mathbf{v}$, etc. We write $S_{\mathbf{u}}$ for the carrier of $\mathbf{u}$, $\leq_{\mathbf{u}}$ for the order of $\mathbf{u}$, and $\lambda_{\mathbf{u}}$ for the labelling of $\mathbf{u}$. We assume that any labelled poset has a carrier that is a subset of some countably infinite set, say $\mathbb{N}$; this allows us to speak about the *set of labelled posets* over $\Sigma$. The precise contents of the carrier, however, are not important — what matters to us is the labels of the points, and the ordering between them.

**Definition 2.2 (Poset isomorphism, pomset).** *Let $\mathbf{u}, \mathbf{v}$ be labelled posets over $\Sigma$. We say $\mathbf{u}$ is* isomorphic *to $\mathbf{v}$, denoted $\mathbf{u} \cong \mathbf{v}$, if there exists a bijection $h : S_{\mathbf{u}} \to S_{\mathbf{v}}$ that preserves labels, and preserves and reflects ordering. More precisely, we require that $\lambda_{\mathbf{v}} \circ h = \lambda_{\mathbf{u}}$, and $s \leq_{\mathbf{u}} s'$ if and only if $h(s) \leq_{\mathbf{v}} h(s')$.*

*A* pomset *over $\Sigma$ is an isomorphism class of labelled posets over $\Sigma$, i.e., the class $[\mathbf{v}] = \{\mathbf{u} : \mathbf{u} \cong \mathbf{v}\}$ for some labelled poset $\mathbf{v}$.*

We write $\mathsf{Pom}(\Sigma)$ for the set of pomsets over $\Sigma$, and 1 for the empty pomset. As long as we have countably many pomsets in scope, the above allows us to assume w.l.o.g. that those pomsets are represented by labelled posets with pairwise disjoint carriers; we tacitly make this assumption throughout this paper.

Pomsets can be concatenated, creating a new pomset that contains all events of the operands, with the same label, but which orders all events of the left operand before those of the right one. We can also compose pomsets in parallel, where events of the operands are juxtaposed without any ordering between them.

**Definition 2.3 (Pomset composition).** *Let $U = [\mathbf{u}]$ and $V = [\mathbf{v}]$ be pomsets over $\Sigma$. We write $U \parallel V$ for the* parallel composition *of $U$ and $V$, which is the pomset over $\Sigma$ represented by the labelled poset $\mathbf{u} \parallel \mathbf{v}$, where*

$$S_{\mathbf{u}\parallel\mathbf{v}} = S_{\mathbf{u}} \cup S_{\mathbf{v}} \qquad \leq_{\mathbf{u}\parallel\mathbf{v}} = \leq_{\mathbf{u}} \cup \leq_{\mathbf{v}} \qquad \lambda_{\mathbf{u}\parallel\mathbf{v}}(x) = \begin{cases} \lambda_{\mathbf{u}}(x) & x \in S_{\mathbf{u}} \\ \lambda_{\mathbf{v}}(x) & x \in S_{\mathbf{v}} \end{cases}$$

*Similarly, we write $U \cdot V$ for the* sequential composition *of $U$ and $V$, that is, the pomset represented by the labelled poset $\mathbf{u} \cdot \mathbf{v}$, where*

$$S_{\mathbf{u}\cdot\mathbf{v}} = S_{\mathbf{u}\parallel\mathbf{v}} \qquad \leq_{\mathbf{u}\cdot\mathbf{v}} = \leq_{\mathbf{u}} \cup \leq_{\mathbf{v}} \cup (S_{\mathbf{u}} \times S_{\mathbf{v}}) \qquad \lambda_{\mathbf{u}\cdot\mathbf{v}} = \lambda_{\mathbf{u}\parallel\mathbf{v}}$$

Just like words are built up from the empty word and letters using concatenation, we can build a particular set of pomsets using only sequential and parallel composition; this will be the primary type of pomset that we will use.

**Definition 2.4 (Series-parallel).** *The set of* series-parallel pomsets *(*sp-pomsets*) over $\Sigma$, denoted $\mathsf{SP}(\Sigma)$, is the smallest set s.t. $1 \in \mathsf{SP}(\Sigma)$, $\mathsf{a} \in \mathsf{SP}(\Sigma)$ for every $\mathsf{a} \in \Sigma$, and it is closed under parallel and sequential composition.*

The following characterisation of $\mathsf{SP}$ is very useful in proofs.

**Theorem 2.5 (Gischer [9]).** *Let $U = [\mathbf{u}] \in \mathsf{Pom}$. Then $U \in \mathsf{SP}$ if and only if $U$ is* N-free*, which is to say that if there exist no distinct $s_0, s_1, s_2, s_3 \in S_{\mathbf{u}}$ such that $s_0 \leq_{\mathbf{u}} s_1$ and $s_2 \leq_{\mathbf{u}} s_3$ and $s_0 \leq_{\mathbf{u}} s_3$, with no other relation between them.*

One way of comparing pomsets is to see whether they have the same events and labels, except that one is "more sequential" in the sense that more events are ordered. This is captured by the notion of *subsumption* [9], defined as follows.

**Definition 2.6 (Subsumption).** *Let $U = [\mathbf{u}]$ and $V = [\mathbf{v}]$. We say $U$ is subsumed by $V$, written $U \sqsubseteq V$, if there exists a label- and order-preserving bijection $h : S_{\mathbf{v}} \to S_{\mathbf{u}}$. That is, $\lambda_{\mathbf{u}} \circ h = \lambda_{\mathbf{v}}$ and if $s \leq_{\mathbf{v}} s'$, then $h(s) \leq_{\mathbf{u}} h(s')$.*

Subsumption between sp-pomsets can be characterised as follows [9].

**Lemma 2.7.** *Let $\sqsubseteq^{\mathsf{sp}}$ be $\sqsubseteq$ restricted to $\mathsf{SP}$. Then $\sqsubseteq^{\mathsf{sp}}$ is the smallest precongruence (preorder monotone w.r.t. the operators) such that for all $U, V, W, X \in \mathsf{SP}$:*

$$(U \parallel V) \cdot (W \parallel X) \sqsubseteq^{\mathsf{sp}} (U \cdot W) \parallel (V \cdot X)$$

**CKA: syntax and semantics.** CKA terms are generated by the grammar

$$e, f \in \mathcal{T}(\Sigma) ::= 0 \mid 1 \mid \mathsf{a} \in \Sigma \mid e + f \mid e \cdot f \mid e \parallel f \mid e^*$$

Semantics of CKA is given in terms of *pomset languages*, that is subsets of $\mathsf{SP}$, which we simply denote by $2^{\mathsf{SP}}$. Formally, the function $[\![-]\!] : \mathcal{T} \to 2^{\mathsf{SP}}$ assigning languages to CKA terms is defined as follows:

$$[\![0]\!] = \emptyset \qquad [\![1]\!] = \{1\} \qquad [\![e + f]\!] = [\![e]\!] \cup [\![f]\!] \qquad [\![e \cdot f]\!] = [\![e]\!] \cdot [\![f]\!]$$
$$[\![e^*]\!] = [\![e]\!]^* \qquad [\![\mathsf{a}]\!] = \{\mathsf{a}\} \qquad [\![e \parallel f]\!] = [\![e]\!] \parallel [\![f]\!]$$

Here, we use the pointwise lifting of sequential and parallel composition from pomsets to pomset languages, i.e., when $\mathcal{U}, \mathcal{V} \subseteq \mathsf{SP}(\Sigma)$, we define

$$\mathcal{U} \cdot \mathcal{V} = \{U \cdot V : U \in \mathcal{U}, V \in \mathcal{V}\} \qquad \mathcal{U} \parallel \mathcal{V} = \{U \parallel V : U \in \mathcal{U}, V \in \mathcal{V}\}$$

Furthermore, the Kleene star of a pomset language $\mathcal{U}$ is defined as $\mathcal{U}^* = \bigcup_{n \in \mathbb{N}} \mathcal{U}^n$, where $\mathcal{U}^0 = \{1\}$ and $\mathcal{U}^{n+1} = \mathcal{U}^n \cdot \mathcal{U}$.

Equivalence of CKA terms can be axiomatised in the style of Kleene algebra. The relation $\equiv$ is the smallest congruence on $\mathcal{T}$ (with respect to all operators) such that for all $e, f, g \in \mathcal{T}$:

$$e + 0 \equiv e \qquad e + e \equiv e \qquad e + f \equiv f + e \qquad e + (f + g) \equiv (f + g) + h$$

$$e \cdot (f \cdot g) \equiv (e \cdot f) \cdot g \qquad e \cdot (f + g) \equiv e \cdot f + e \cdot h \qquad (e + f) \cdot g \equiv e \cdot g + f \cdot g$$

$$e \cdot 1 \equiv e \equiv 1 \cdot e \qquad e \cdot 0 \equiv 0 \equiv 0 \cdot e \qquad e \parallel f \equiv f \parallel e \qquad e \parallel 1 \equiv e \qquad e \parallel 0 \equiv 0$$

$$e \parallel (f \parallel g) \equiv (e \parallel f) \parallel g \qquad e \parallel (f + g) \equiv e \parallel f + e \parallel g \qquad 1 + e \cdot e^* \equiv e^* \equiv 1 + e^* \cdot e$$

$$e + f \cdot g \leqq g \implies f^* \cdot e \leqq g \qquad e + f \cdot g \leqq f \implies e \cdot g^* \leqq f$$

in which $e \leqq f$ is the natural order $e + f \equiv f$. The final (conditional) axioms are referred to as the *least fixpoint axioms*.

Laurence and Struth [23] proved this axiomatisation to be sound and complete. A decision procedure was proposed in [4].

**Theorem 2.8 (Soundness, completeness, decidability).** *Let $e, f \in \mathcal{T}$. We have: $e \equiv f$ if and only if $[\![e]\!] = [\![f]\!]$, and it is decidable whether $[\![e]\!] = [\![f]\!]$.*

Readers familiar with CKA will notice that the algebra defined here is not in fact CKA as defined in [11]. Indeed the signature axiom of CKA, the exchange law, has been omitted. However, as we show in Section 4.2, the standard definition of CKA, as well as its completeness proof [15], may be recovered using hypotheses.

## 3 Pomset contexts

The linear one-dimensional structure of words makes it straightforward to define occurrences of subwords: if one wants to state that a word $w$ appears in another word $v$, one can simply say that $v = xwy$ for some $x$ and $y$. Due to the two-dimensional nature of pomsets, it is not straightforward to define when a pomset occurs inside another pomset, because the pomset could appear below a parallel, which is nested in a sequential, which is in a parallel, etc. In what follows we define *pomset contexts*, that will enable us to talk about pomset factorisations in a similar fashion as we do for words, and prove some useful properties for these.

**Definition 3.1.** *Let $*$ be a symbol not occurring in $\Sigma$. A* pomset context *is a pomset over $\Sigma \cup \{*\}$ with exactly* one *node labelled by $*$. More precisely, $C$ is a pomset context if $C = [\mathbf{c}]$ with exactly one $s_* \in S_\mathbf{c}$ with $\lambda_\mathbf{c}(s_*) = *$.*

Intuitively, $*$ is a placeholder or gap where another pomset can be inserted. We write $\mathsf{PC}(\Sigma)$ for the set of pomset contexts over $\Sigma$, and $\mathsf{PC}^{\mathsf{sp}}(\Sigma)$ for the series-parallel pomset contexts over $\Sigma$.

Given a $C \in \mathsf{PC}$ and $U \in \mathsf{Pom}$, we can "plug" $U$ into the gap left in $C$ to obtain the pomset $C[U] \in \mathsf{Pom}$. More precisely, let $U = [\mathbf{u}]$ and $C = [\mathbf{c}]$ with $\mathbf{u}$ disjoint from $\mathbf{c}$. We write $C[U]$ for the pomset represented by $\mathbf{c}[\mathbf{u}]$, where $S_{\mathbf{c}[\mathbf{u}]} = S_\mathbf{u} \cup S_\mathbf{c} - \{*\}$ and $\lambda_{\mathbf{c}[\mathbf{u}]}(s)$ is given by $\lambda_\mathbf{c}(s)$ if $s \in S_\mathbf{c} - \{*\}$, and $\lambda_\mathbf{u}(s)$ when $s \in S_\mathbf{u}$; lastly, $\leq_{\mathbf{c}[\mathbf{u}]}$ is the smallest relation on $S_{\mathbf{c}[\mathbf{u}]}$ satisfying

$$\frac{s \leq_\mathbf{u} s'}{s \leq_{\mathbf{c}[\mathbf{u}]} s'} \qquad \frac{s \leq_\mathbf{c} s'}{s \leq_{\mathbf{c}[\mathbf{u}]} s'} \qquad \frac{s_* \leq_\mathbf{c} s \qquad s' \in S_\mathbf{u}}{s' \leq_{\mathbf{c}[\mathbf{u}]} s} \qquad \frac{s' \in S_\mathbf{u} \qquad s \leq_\mathbf{c} s_*}{s \leq_{\mathbf{c}[\mathbf{u}]} s'}$$

It follows easily that $\leq_{\mathbf{c}[\mathbf{u}]}$ is a partial order. We may also apply contexts to languages: if $L \subseteq \mathsf{Pom}$ and $C \in \mathsf{PC}$, the language $C[L]$ is defined as $\{C[U] : U \in L\}$.

We now prove some properties of contexts that will be useful later in our technical development. First, we note that pomset contexts respect subsumption.

**Lemma 3.2.** *Let $C, D \in \mathsf{PC}$, $U \in \mathsf{Pom}$. If $C \sqsubseteq D$, then $C[U] \sqsubseteq D[U]$.*

Series-parallel pomset contexts can be given an inductive characterisation.

**Lemma 3.3.** $\mathsf{PC}^{\mathsf{sp}}$ *is the smallest pomset language $L$ satisfying*

$$\frac{}{* \in L} \qquad \frac{U \in \mathsf{SP} \qquad C \in L}{U \cdot C \in L} \qquad \frac{C \in L \qquad V \in \mathsf{SP}}{C \cdot V \in L} \qquad \frac{U \in \mathsf{SP} \qquad C \in L}{U \parallel C \in L}$$

We will identify *totally ordered pomsets* with words, i.e., $\Sigma^* \subseteq \mathsf{SP}$. If the pomset $U$ inserted in a context $C$ is a non-empty word, and the resulting pomset is a parallel pomset, then we can infer how to factorise $C$.

**Lemma 3.4.** *Let $C \in \mathsf{PC}^{\mathsf{sp}}$ be a pomset context, let $V, W \in \mathsf{Pom}$, and let $U \in \Sigma^*$ be non-empty. If $C[U] = V \parallel W$, then there exists a $C' \in \mathsf{PC}^{\mathsf{sp}}$ such that either $C = C' \parallel W$ and $C'[U] = V$, or $C = V \parallel C'$ and $C'[U] = W$.*

Application of series-parallel contexts preserves series-parallel pomsets.

**Lemma 3.5.** *Let $C \in \mathsf{PC}^{\mathsf{sp}}$. If $U \in \mathsf{SP}$, then $C[U] \in \mathsf{SP}$ as well.*

If we plug the empty pomset into a context, then any subsumed pomset can be obtained by plugging the empty pomset into a subsumed context. If the subsumed pomset is series-parallel, then so is the subsumed context.

**Lemma 3.6.** *Let $C \in \mathsf{PC}$ and $V \in \mathsf{Pom}$ with $V \sqsubseteq C[1]$. We can construct $C' \in \mathsf{PC}$ such that $C' \sqsubseteq C$ and $C'[1] = V$. Moreover, if $V \in \mathsf{SP}$, then $C' \in \mathsf{PC}^{\mathsf{sp}}$.*

An analogue to the previous lemma can be obtained if instead of the empty pomset one inserts a single letter pomset $a$.

**Lemma 3.7.** *Let $C \in \mathsf{PC}$, $V \in \mathsf{Pom}$ and $\mathsf{a} \in \Sigma$ with $V \sqsubseteq C[\mathsf{a}]$. We can construct $C' \in \mathsf{PC}$ s.t. $C' \sqsubseteq C$ and $C'[\mathsf{a}] = V$. Moreover, if $V \in \mathsf{SP}$, then $C' \in \mathsf{PC}^{\mathsf{sp}}$.*

## 4 Concurrent Kleene Algebra with Hypotheses

Kleene algebra has basic axioms about how program composition operators should work in general, and hence does not make any assumptions about how these operators work on specific programs. When reasoning about equivalence in a programming language, however, it makes sense to embed domain-specific truths about the operators into the axioms. For instance, if a programming language includes assignments to variables, then subsequent assignments to the same variable could be merged into one, giving rise to an equation such as

$$x \leftarrow m \leq x \leftarrow n \cdot x \leftarrow m, \tag{1}$$

which says that the behaviour of first assigning $n$, then $m$ to $x$ (on the right) includes the behaviour of simply assigning $m$ to $x$ directly (on the left).

Kleene algebra with hypotheses (KAH) [5,18,20,7] enables the addition of extra axioms, called *hypotheses*, to the axioms of KA. The appeal of KAH is that it allows a wide range of such hypotheses about programs to be added to the equational theory, while retaining the theoretical boilerplate of KA. In particular, it turns out that we can derive a sound model for any set of hypotheses, using the language model that is sound for KA proper [7]. Moreover, the completeness and decidability results that hold for KA can be leveraged to obtain completeness and decidability results for some specific types of hypotheses [5,20,7]; in general, equivalence under other hypotheses may turn out to be undecidable [18].

In this section, we propose a generalisation of so-called Kleene algebra with hypotheses to a concurrent setting, showing how one can obtain a sound (pomset language) model for any set of hypotheses. We then discuss a number of techniques that allow one to prove completeness and decidability of the resulting system for a large set of hypotheses, by relying on analogous results about CKA.

**Definition 4.1.** *A* hypothesis *is an inequation $e \leq f$ where $e, f \in \mathcal{T}$. When $H$ is a set of hypotheses, we write $\equiv^H$ for the smallest congruence on $\mathcal{T}$ generated by the hypotheses in $H$ as well as the axioms and implications that build $\equiv$. More concretely, whenever $e \leq f \in H$, also $e \leqq^H f$.*

A hypothesis that declares two programs to be equivalent, such as in (1), can be encoded by including both $e \leq f$ and $f \leq e$ in $H$.

*Example 4.2.* Suppose the set of primitive actions $\Sigma$ includes the increments of the form $\mathtt{incr}\,x$, as well as a statement $\mathtt{print}$, which writes the complete state of the machine (including variables) on the standard output. Since we would like to depict the state consistently, the state should not change while the output is rendered; hence, $\mathtt{print}$ cannot be executed concurrently with any other action. Instead, when a program containing $\mathtt{print}$ is scheduled to run in parallel with an assignment, it must be interleaved such that the assignment runs either entirely before or after $\mathtt{print}$. To encode this, we can include in $H$ the hypotheses

$$\mathtt{incr}\,x \parallel \mathtt{print} = \mathtt{incr}\,x \cdot \mathtt{print} + \mathtt{print} \cdot \mathtt{incr}\,x$$

for all variables $x$. This allows us to prove, for instance, that

$$\mathtt{print} \cdot \mathtt{incr}\,x \cdot \mathtt{incr}\,x \cdot \mathtt{print} \leqq^H (\mathtt{incr}\,x \parallel \mathtt{print})^*$$

That is, if we run some number of increments and $\mathtt{print}$ statements in parallel, it is possible that $x$ is incremented twice between print statements.

To obtain a model of CKAH, it is not enough to use $[\![-]\!]$, as some programs equated by the hypotheses might have different semantics. To get around this, we adapt the method from [7]: take $[\![-]\!]$ as a base semantics, and adapt the resulting language using hypotheses, such that the pomsets that could be obtained by rearranging the term using the hypotheses are also present in the language:

**Definition 4.3.** *Let $L \subseteq \mathsf{Pom}$. We define the $H$-closure of $L$, written $L{\downarrow}^H$, as the smallest language containing $L$ such that for all $e \leq f \in H$ and $C \in \mathsf{PC}^{\mathsf{sp}}$, if $C[[\![f]\!]] \subseteq L{\downarrow}^H$, then $C[[\![e]\!]] \subseteq L{\downarrow}^H$. Formally, $L{\downarrow}^H$ may be described as the smallest language satisfying the following inference rules:*

$$\frac{}{L \subseteq L{\downarrow}^H} \qquad \frac{e \leq f \in H \qquad C \in \mathsf{PC}^{\mathsf{sp}} \qquad C[[\![f]\!]] \subseteq L{\downarrow}^H}{C[[\![e]\!]] \subseteq L{\downarrow}^H}$$

*Example 4.4.* Continuing with $H$ and $\Sigma$ as in the previous examples, note that if $L = [\![\mathtt{incr}\,x \parallel \mathtt{print}]\!]$, then $\mathtt{incr}\,x \parallel \mathtt{print} \in L{\downarrow}^H$. Choose $C = *$; we have $C[\mathtt{incr}\,x \cdot \mathtt{print}] = \mathtt{incr}\,x \cdot \mathtt{print}$. Because $\mathtt{incr}\,x \cdot \mathtt{print} + \mathtt{print} \cdot \mathtt{incr}\,x \leq \mathtt{incr}\,x \parallel \mathtt{print} \in H$ and for all $U \in [\![\mathtt{incr}\,x \parallel \mathtt{print}]\!]$ we have $C[U] \in L \subseteq L{\downarrow}^H$, we get $C[\mathtt{incr}\,x \cdot \mathtt{print}] \in L{\downarrow}^H$ and therefore $\mathtt{incr}\,x \cdot \mathtt{print} \in L{\downarrow}^H$.

We observe the following useful properties about the interaction between closure and other operators on pomset languages.

**Lemma 4.5.** *Let $L, K \subseteq \mathsf{Pom}$ and $C \in \mathsf{PC}^{\mathsf{sp}}$. The following hold.*

1. $L \subseteq K{\downarrow}^H$ *iff* $L{\downarrow}^H \subseteq K{\downarrow}^H$.
2. *If* $L \subseteq K$, *then* $L{\downarrow}^H \subseteq K{\downarrow}^H$.
3. $(L \cup K){\downarrow}^H = \left(L{\downarrow}^H \cup K{\downarrow}^H\right){\downarrow}^H$
4. $(L \cdot K){\downarrow}^H = \left(L{\downarrow}^H \cdot K{\downarrow}^H\right){\downarrow}^H$
5. $(L \parallel K){\downarrow}^H = \left(L{\downarrow}^H \parallel K{\downarrow}^H\right){\downarrow}^H$
6. $(L^*){\downarrow}^H = \left(\left(L{\downarrow}^H\right)^*\right){\downarrow}^H$
7. *If* $L{\downarrow}^H \subseteq K{\downarrow}^H$, *then* $C[L]{\downarrow}^H \subseteq C[K]{\downarrow}^H$.
8. *If* $L \subseteq \mathsf{SP}$, *then* $L{\downarrow}^H \subseteq \mathsf{SP}$.

*Remark 4.6.* Property (1) states that $-{\downarrow}^H$ is a closure operator. However, it is not in general a Kuratowski closure operator [22], since it fails to commute with union. For instance, let $\mathtt{a}, \mathtt{b}, \mathtt{c} \in \Sigma$ and $H = \{\mathtt{a} \leq \mathtt{b} + \mathtt{c}\}$; then $\{\mathtt{b}\}{\downarrow}^H \cup \{\mathtt{c}\}{\downarrow}^H = \{\mathtt{b}, \mathtt{c}\}$, while $\mathtt{a} \in (\{\mathtt{b}\} \cup \{\mathtt{c}\}){\downarrow}^H$.

Using Lemma 4.5, we can show that, if we combine the semantics from $[\![-]\!]$ with $H$-closure, we obtain a sound semantics for CKA with hypotheses $H$.

**Lemma 4.7 (Soundness).** *If $e \equiv^H f$, then $[\![e]\!]{\downarrow}^H = [\![f]\!]{\downarrow}^H$.*

The converse of the above, where semantic equivalence is sufficient to establish axiomatic equivalence, is called *completeness*. Similarly, we may also be interested in *deciding* whether $[\![e]\!]{\downarrow}^H$ and $[\![f]\!]{\downarrow}^H$ coincide.

**Definition 4.8.** *Let $e, f \in \mathcal{T}$.*

*(i) If $[\![e]\!]{\downarrow}^H = [\![f]\!]{\downarrow}^H$ implies $e \equiv^H f$, then $H$ is called* complete.
*(ii) If $[\![e]\!]{\downarrow}^H = [\![f]\!]{\downarrow}^H$ is decidable, then $H$ is said to be* decidable.

Note that, in the special case where $H = \emptyset$, we know that $H$ is complete and decidable by Theorem 2.8. One method to find out whether $H$ is complete or decidable is to reduce the problem to this special case. More concretely, suppose we know $[\![e]\!]{\downarrow}^H = [\![f]\!]{\downarrow}^H$, and want to establish that $e \equiv^H f$. If we could find a set of hypotheses $H'$ that is complete, and we could map $e$ and $f$ to terms $r(e)$ and $r(f)$ such that $[\![r(e)]\!]{\downarrow}^{H'} = [\![r(f)]\!]{\downarrow}^{H'}$, then we would have $r(e) \equiv^{H'} r(f)$. If we could then "lift" that equivalence to prove $e \equiv^H f$, we are done. Similarly, if we would know that $[\![r(e)]\!]{\downarrow}^{H'} = [\![r(f)]\!]{\downarrow}^{H'}$ is equivalent to $[\![e]\!]{\downarrow}^H = [\![f]\!]{\downarrow}^H$, we could decide the latter. To formalise this intuition, we first need the following.

**Definition 4.9.** *We say that $H$* implies *$H'$ if we can use the hypotheses in $H$ to prove those of $H'$, i.e., if for every hypothesis $e \leq f \in H'$ it holds that $e \leqq^H f$.*

Implication relates to equivalence and closure as follows.

**Lemma 4.10.** *Let $H$ and $H'$ be sets of hypotheses such that $H$ implies $H'$.*

*(i) If $e, f \in \mathcal{T}$ with $e \equiv^{H'} f$, then $e \equiv^H f$.*
*(ii) If $L \subseteq \mathsf{Pom}$, then $L{\downarrow}^{H'} \subseteq L{\downarrow}^H$.*
*(iii) If $L \subseteq \mathsf{Pom}$, then $(L{\downarrow}^{H'}){\downarrow}^H = L{\downarrow}^H$.*

If $H$ implies $H'$ and vice versa, then $H$ is complete (resp. decidable) precisely when $H'$ is. In general, however, this is not very helpful; we need something more asymmetrical, in order to get from a complicated set of hypotheses $H$ to a simpler set of hypotheses $H'$, where completeness or decidability might be easier to prove. Ideally, we would like to reduce to $H' = \emptyset$, which is complete and decidable.

One idea to formalise this idea of a reduction is as follows.

**Definition 4.11.** *Let $H$ and $H'$ be sets of hypotheses such that $H$ implies $H'$. A map $r : \mathcal{T} \to \mathcal{T}$ is a* reduction *from $H$ to $H'$ when both of the following are true:*

*(i) for $e \in \mathcal{T}$, it holds that $e \equiv^H r(e)$, and*
*(ii) for $e, f \in \mathcal{T}$, if $[\![e]\!]{\downarrow}^H = [\![f]\!]{\downarrow}^H$, then $[\![r(e)]\!]{\downarrow}^{H'} = [\![r(f)]\!]{\downarrow}^{H'}$.*

*We call $H$* reducible *to $H'$ if there exists a reduction from $H$ to $H'$.*

It is straightforward to show that reductions do indeed carry over completeness and decidability results, in the following sense.

**Lemma 4.12.** *Suppose $H$ is reducible to $H'$. If $H'$ is complete (respectively decidable), then so is $H$.*

*Example 4.13.* Let $\Sigma = \{\mathtt{a}, \mathtt{b}\}$. Let $H = \{\mathtt{a} \leq \mathtt{b}\}$. We can define for $e \in \mathcal{T}$ the term $r(e) \in \mathcal{T}$, which is $e$ but with every occurrence of $\mathtt{b}$ replaced by $\mathtt{a} + \mathtt{b}$. For instance, $r(\mathtt{a} \cdot \mathtt{b}^* \parallel \mathtt{c}) = \mathtt{a} \cdot (\mathtt{a} + \mathtt{b})^* \parallel \mathtt{c}$. An inductive argument on the structure of $e$ shows that $r$ reduces $H$ to $\emptyset$, and hence $H$ is complete and decidable.

It is not very hard to show that reductions can be chained, as follows.

**Lemma 4.14.** *If $H$ reduces to $H'$, which reduces to $H''$, then $H$ reduces to $H''$.*

Another way of reducing $H$ is to find two sets of hypotheses $H_0$ and $H_1$, and reduce each of those to another set of hypotheses $H'$ [7]. The idea is that a proof of $e \equiv^H f$ can be split up in a phase where we find $e', f' \in \mathcal{T}$ such that $e \equiv^{H_0} e'$ and $f \equiv^{H_0} f'$, after which we find $e'', f'' \in \mathcal{T}$ with $e' \equiv^{H_1} e''$ and $f' \equiv^{H_1} f''$. Finally, we establish that $e'' \equiv^{H'} f''$, before lifting those equivalences to $H$, concluding

$$e \equiv^H e' \equiv^H e'' \equiv^H f'' \equiv^H f' \equiv^H f$$

One way of achieving this is as follows.

**Definition 4.15.** *We say that $H$* factorises *into $H_0$ and $H_1$ if $H$ implies both $H_0$ and $H_1$, and for all $L \subseteq \mathsf{SP}$ we have that $L{\downarrow}^H = (L{\downarrow}^{H_0}){\downarrow}^{H_1}$.*

In order to use factorisation to compose simpler reductions into more complicated ones, we need a slightly stronger notion of reduction, as follows.

**Definition 4.16.** *We say that $r$ is a* strong reduction *from $H$ to $H'$ if it is a reduction such that for $e \in \mathcal{T}$, it holds that $[\![e]\!]{\downarrow}^H = [\![r(e)]\!]{\downarrow}^{H'}$.*

Note that this additional condition essentially strengthens the second condition in Definition 4.11. Factorisation then lets us compose strong reductions.

**Lemma 4.17.** *Suppose $H$ factorises into $H_0$ and $H_1$, and both $H_0$ and $H_1$ strongly reduce to $H'$. Then $H$ strongly reduces to $H'$.*

The remainder of this section is devoted to developing techniques that can be used to design reductions, based on the properties of the sets of hypotheses under consideration. Using the lemmas we have established so far, these techniques may then be leveraged to obtain completeness and decidability results.

## 4.1 Reification

It can happen that the hypotheses in $H$ impose an algebraic structure on the letters in $\Sigma$; for instance, as we will see later on, the letters in $H$ could be propositional terms, whose equivalence is mediated by the axioms of Boolean algebra. In order to peel away this layer of axioms and reduce to a smaller $H'$, we can try to reduce to terms over a smaller alphabet, making the algebraic structure on the letters irrelevant to equivalence. In a sense, performing this kind of reduction is like showing that the equivalences between letters from the hypotheses can already be guaranteed by replacing them with the right terms.

*Example 4.18.* Let $\Sigma$ be the set of group terms over a (finite) alphabet $\Lambda$, that is, $\Sigma$ consists of the terms generated by the grammar $g, h ::= u \mid \mathtt{a} \in \Lambda \mid g \circ h \mid \overline{g}$. Furthermore, let $\equiv_G$ be the smallest congruence generated by the group axioms, i.e., for all $g, h, i \in \Lambda$ it holds that

$$g \circ (h \circ i) \equiv_G (g \circ h) \circ i \qquad g \circ u \equiv_G g \equiv_G u \circ g \qquad \overline{g} \circ g \equiv_G u \equiv_G g \circ \overline{g}$$

Lastly, let $\mathsf{group} = \{g \leq h : g \equiv_G h\}$. We can then define a reduction from $\mathsf{group}$ to $\emptyset$ by replacing every letter (group term) in a term $e$ with its reduced form, that is, with the (unique) equivalent group term of minimum size. For instance, if $\Lambda = \{\mathtt{a}, \mathtt{b}, \mathtt{c}\}$, then we send the term $\mathtt{a} \circ \overline{\mathtt{a}} \parallel \mathtt{b} \circ \mathtt{c} \circ \overline{\mathtt{c}}$ to the term $u \parallel \mathtt{b}$.

For the remainder of this section, we fix a subalphabet $\Gamma \subseteq \Sigma$. When $r : \Sigma \to \mathcal{T}(\Gamma)$, we extend $r$ to a map from $\mathcal{T}(\Sigma)$ to $\mathcal{T}(\Gamma)$, by inductively applying $r$ to terms. We can also apply $r$ to a series-parallel pomset, obtaining a pomset language. More precisely, when $U$ is a pomset, we define $r(U)$ as follows:

$$r(1) = \{1\} \quad r(U \cdot V) = r(U) \cdot r(V) \quad r(\mathtt{a}) = [\![ r(\mathtt{a}) ]\!] \quad r(U \parallel V) = r(U) \parallel r(V)$$

Lastly, when $L \subseteq \mathsf{SP}$, we write $r(L)$ for the set $\bigcup\{r(U) : U \in L\}$.

The following then formalises the idea of reducing by replacing letters.

**Definition 4.19.** *A map $r : \Sigma \to \mathcal{T}(\Gamma)$ is a* reification *from $H$ to $H'$ if*

*(i) For all $\mathtt{a} \in \Sigma$, it holds that $r(\mathtt{a}) \equiv^H \mathtt{a}$.*
*(ii) $r$ is expansive on $\Gamma$, i.e., for all $\mathtt{a} \in \Gamma$, $\mathtt{a} \leqq r(\mathtt{a})$.*
*(iii) $H'$-closure preserves $\Gamma$, i.e., for all $L \subseteq \mathsf{SP}(\Gamma)$, also $L{\downarrow}^{H'} \subseteq \mathsf{SP}(\Gamma)$.*
*(iv) For all $e \leq f \in H$, it holds that $r(e) \leqq^{H'} r(f)$.*

*Example 4.20.* Continuing with the previous example, let $r$ be the map that sends a group term to its reduced form; we claim that $r$ is a reification from $\mathsf{group}$ to $\emptyset$. By definition, we then know that for a group term $g \in \Sigma$, we have $r(g) \equiv_G g$, and hence $r(g) \equiv^{\mathsf{group}} g$. Furthermore, the reduction of a reduced term is that term itself; hence, the second condition is satisfied. The third condition holds trivially. Lastly, if $e \leq f \in \mathsf{group}$, then $e, f \in \Sigma$ such that $e \equiv_G f$. Since reductions are unique, we then know that $r(e) = r(f)$, and hence $r(e) \leqq^{\emptyset} r(f)$.

We have the following general properties of a map $r$, which we will use in demonstrating how to obtain a reduction from a reification.

**Lemma 4.21.** *Let $r : \Sigma \to \mathcal{T}$ be some map.*

*(i) For all $C \in \mathsf{PC}^{\mathsf{sp}}$, we have $r(C) \subseteq \mathsf{PC}^{\mathsf{sp}}$.*
*(ii) For all $L \subseteq \mathsf{SP}$ and $C \in \mathsf{PC}^{\mathsf{sp}}$, we have $r(C[L]) = \bigcup_{D \in r(C)} D[r(L)]$.*
*(iii) For all $e \in \mathcal{T}$, it holds that $r(\llbracket e \rrbracket) = \llbracket r(e) \rrbracket$.*

The following technical lemma is a consequence of property (iv).

**Lemma 4.22.** *If $r$ is a reification and $L \subseteq \mathsf{SP}(\Sigma)$, then $r(L{\downarrow}^H) \subseteq r(L){\downarrow}^{H'}$.*

Using this, we can then show how to obtain a reduction from a reification.

**Lemma 4.23.** *If $H$ implies $H'$ and $r$ is a reification from $H$ to $H'$, then $r$ is a reduction from $H$ to $H'$.*

*Proof.* The first condition, i.e., that for $e \in \mathcal{T}$ we have $e \equiv^H r(e)$, can be checked using the first property of reification by induction on the structure of $e$. It thus remains to check the second condition; we do this by proving that for all $e \in \mathcal{T}(\Sigma)$ we have $r\left(\llbracket e \rrbracket{\downarrow}^H\right) = \llbracket r(e) \rrbracket{\downarrow}^{H'}$. To this end, we derive as follows:

$$\begin{aligned} r(\llbracket e \rrbracket{\downarrow}^H) &\subseteq r(\llbracket e \rrbracket){\downarrow}^{H'} && \text{(Lemma 4.22)} \\ &= \llbracket r(e) \rrbracket{\downarrow}^{H'} && \text{(Lemma 4.21(iii))} \\ &\subseteq r(\llbracket r(e) \rrbracket{\downarrow}^{H'}) && \text{(property (ii))} \\ &\subseteq r(\llbracket r(e) \rrbracket{\downarrow}^{H}) && \text{(Lemma 4.10(ii))} \\ &= r(\llbracket e \rrbracket{\downarrow}^H) && \text{(property (i), soundness)} \end{aligned}$$

Specifically, in the third step, property (ii) ensures that for $L \subseteq \mathsf{SP}(\Gamma)$ we have $L \subseteq r(L)$. We can use this property because $H'$-closure preserves the $\Gamma$-language by property (iii). This completes the proof. □

## 4.2 Factoring the exchange law

In the basic axioms that generate $\equiv$, there is no interaction between sequential and parallel composition. One sensible way of adding that kind of interaction is, as suggested by Hoare, Struth and collaborators [11], by adding an axiom of the form $(e \parallel f) \cdot (g \parallel h) \leqq (e \cdot g) \parallel (f \cdot h)$, known as the *exchange law*. Essentially, this axiom encodes the possibility of (partial) interleaving: when $e \cdot g$ runs in parallel with $f \cdot h$, one possible behaviour is that, first $e$ runs in parallel with $f$, and then $g$ runs in parallel with $h$. The core observation of this section is that the exchange law can be treated as another set of hypotheses, as we show below, and this can then be used to recover the completeness result of CKA [15].

**Definition 4.24.** *We write* $\mathsf{exch}$ *for the set*

$$\{(e \parallel f) \cdot (g \parallel h) \leq (e \cdot g) \parallel (f \cdot h) : e, f, g, h \in \mathcal{T}\}$$

The semantic effect of adding $\mathsf{exch}$ to our hypotheses is that, if $U$ is a pomset in a series-parallel language $L$, and $V$ is a series-parallel pomset subsumed by $U$, then $V$ is in the $\mathsf{exch}$-closure of $L$. Intuitively, the $\mathsf{exch}$-closure adds pomsets that are more sequential, i.e., have more ordering, than the ones already in $L$. Indeed, $\mathsf{exch}$-closure coincides with the downward closure w.r.t. $\sqsubseteq^{\mathsf{sp}}$.

**Lemma 4.25.** *Let* $L \subseteq \mathsf{SP}$ *and* $U \in \mathsf{SP}$. *Now* $U \in L{\downarrow}^{\mathsf{exch}}$ *if and only if there exists a* $V \in L$ *such that* $U \sqsubseteq^{\mathsf{sp}} V$.

We have previously shown that $\mathsf{exch}$ is complete [15]; as a matter of fact, the pivotal result from op. cit. can be presented as follows.

**Theorem 4.26.** *The set of hypotheses* $\mathsf{exch}$ *is strongly reducible to* $\emptyset$.

When $\mathsf{exch}$ is contained in our hypotheses, it is not immediately clear whether those hypotheses can be reduced. What we can do is try to factorise our hypotheses into $\mathsf{exch}$ and some residual set of hypotheses, and prove strong reducibility for that residual set. To this end, we first note that, in some circumstances, the $H$-closure of the $\mathsf{exch}$-closure remains downward-closed w.r.t. $\sqsubseteq^{\mathsf{sp}}$.

**Lemma 4.27.** *Suppose that for each* $e \leq f \in H$ *we have that* $e = 1$ *or* $e = \mathsf{a}$ *for some* $\mathsf{a} \in \Sigma$, *and let* $L \subseteq \mathsf{SP}$. *If* $U, V \in \mathsf{SP}$ *such that* $U \sqsubseteq^{\mathsf{sp}} V$ *and* $V \in (L{\downarrow}^{\mathsf{exch}}){\downarrow}^{H}$, *then* $U \in (L{\downarrow}^{\mathsf{exch}}){\downarrow}^{H}$.

Using this fact, we can now show that, under the same precondition, $\mathsf{exch} \cup H$ factors into $\mathsf{exch}$ and $H$. This factorisation is what we were looking for: it tells us that whenever $H$ strongly reduces to $\emptyset$, so does $H \cup \mathsf{exch}$.

**Lemma 4.28.** *Suppose that for each* $e \leq f \in H$ *we have that* $e = 1$, *or* $e = \mathsf{a}$ *for some* $\mathsf{a} \in \Sigma$. *Then* $H \cup \mathsf{exch}$ *factorises into* $\mathsf{exch}$ *and* $H$.

*Proof.* Since $H, \mathsf{exch} \subseteq H \cup \mathsf{exch}$, it should be obvious that $H \cup \mathsf{exch}$ implies both $H$ and $\mathsf{exch}$. It remains to show that, if $L \subseteq \mathsf{SP}$, then $(L{\downarrow}^{\mathsf{exch}}){\downarrow}^{H} = L{\downarrow}^{H \cup \mathsf{exch}}$. The inclusion from left to right is a consequence of Lemma 4.10(ii)–(iii).

For the other inclusion, we show that if $A \subseteq L{\downarrow}^{H \cup \mathsf{exch}}$, then $A \subseteq (L{\downarrow}^{\mathsf{exch}}){\downarrow}^{H}$. The proof proceeds by induction on the construction of $A \subseteq L{\downarrow}^{H \cup \mathsf{exch}}$. In the base, we have that $A \subseteq L{\downarrow}^{H \cup \mathsf{exch}}$ because $A = L$; in that case, $A \subseteq L{\downarrow}^{\mathsf{exch}} \subseteq (L{\downarrow}^{\mathsf{exch}}){\downarrow}^{H}$.

For the inductive step, $A \subseteq L{\downarrow}^{H \cup \mathsf{exch}}$ because there exist $e \leq f \in H \cup \mathsf{exch}$ and $C \in \mathsf{PC}^{\mathsf{sp}}$ such that $A = C[\llbracket e \rrbracket]$, and $C[\llbracket f \rrbracket] \subseteq L{\downarrow}^{H \cup \mathsf{exch}}$. By induction, we then know that $C[\llbracket f \rrbracket] \subseteq \left(L{\downarrow}^{\mathsf{exch}}\right){\downarrow}^{H}$. On the one hand, if $e \leq f \in H$, then $A = C[\llbracket e \rrbracket] \subseteq \left(L{\downarrow}^{\mathsf{exch}}\right){\downarrow}^{H}$ immediately. On the other hand, if $e \leq f \in \mathsf{exch}$, then $\llbracket e \rrbracket \sqsubseteq^{\mathsf{sp}} \llbracket f \rrbracket$, and hence $C[\llbracket e \rrbracket] \sqsubseteq^{\mathsf{sp}} C[\llbracket f \rrbracket]$ by Lemma 3.2. By Lemma 3.5 and Lemma 4.27, it then follows that $A = C[\llbracket e \rrbracket] \subseteq (L{\downarrow}^{\mathsf{exch}}){\downarrow}^{H}$. □

### 4.3 Lifting

A number of reduction procedures already exist at the level of Kleene algebra [20,7]; ideally, one would like to lift those procedures to CKA.

*Example 4.29.* The reductions in Example 4.13 and Example 4.18 worked out for terms without $\parallel$, and then extended inductively, by defining the reduction of $e \parallel f$ to be the parallel composition of the reductions of $e$ and $f$ respectively.

As a non-example, consider $H = \{\mathtt{a} \leq 1\}$. Even though this hypothesis can be reduced to $\emptyset$ within Kleene algebra [5], it is not obvious how this would work for pomset languages. In particular, if $1 \in L$, then $1 \parallel \cdots \parallel 1 \in L$ for any number of 1's, and hence $\mathtt{a} \parallel \cdots \parallel \mathtt{a} \in L{\downarrow}^H$ for any number of $\mathtt{a}$'s. This precludes the possibility of a strong reduction to $\emptyset$, because $[\![1]\!]{\downarrow}^H$ is a pomset language of unbounded (parallel) width, which cannot be expressed by any $e \in \mathcal{T}$ [25].

We now establish a set of sufficient conditions for such a lifting to work. To this end, we first formally define Kleene algebra syntax, axioms and semantics.

**Definition 4.30.** *Write $\mathcal{T}_{\mathsf{KA}}$ for the set of* Kleene algebra terms*, i.e., the terms in $\mathcal{T}$ that do not contain $\parallel$. Furthermore, we write $\equiv_{\mathsf{KA}}$ for the smallest congruence on $\mathcal{T}_{\mathsf{KA}}$ that is generated by the axioms of $\equiv$ that do not involve $\parallel$.*

When $e \in \mathcal{T}_{\mathsf{KA}}$, it is not hard to see that $[\![e]\!]$ contains totally ordered pomsets, i.e., words, exclusively. Using these definitions, we can now specialise the notions of hypotheses, context, and closure to the sequential setting, as follows.

**Definition 4.31.** *The relation $\equiv^H_{\mathsf{KA}}$ is generated from $H$ and $\equiv_{\mathsf{KA}}$ as before.*

*A context $C \in \mathsf{PC}^{\mathsf{sp}}$ is* sequential *if it is totally ordered, i.e., if it is a word with one occurrence of $*$; we write $\mathsf{PC}^{\mathsf{seq}}$ for the set of sequential contexts.*

*Given a set of hypotheses $H$ and a language $L \subseteq \Sigma^*$, we define the* sequential closure *of $L$ with respect to $H$, written $L{\downarrow}^H_{\mathsf{seq}}$, as the least language containing $L$ such that for all $e \leq f \in H$ and $C \in \mathsf{PC}^{\mathsf{seq}}$, if $C[[\![f]\!]] \subseteq L{\downarrow}^H_{\mathsf{seq}}$, then $C[[\![e]\!]] \subseteq L{\downarrow}^H_{\mathsf{seq}}$.*

If $\parallel$ does not occur in any hypothesis, then the definition of sequential closure coincides with the closure operator from [7]. Thus, if $L \subseteq \Sigma^*$, then $L{\downarrow}^H_{\mathsf{seq}} \subseteq \Sigma^*$.

The analogue of strong reduction for the sequential setting is as follows.

**Definition 4.32.** *Suppose that $H$ implies $H'$. A map $r : \mathcal{T}_{\mathsf{KA}} \to \mathcal{T}_{\mathsf{KA}}$ is a* sequential reduction *from $H$ to $H'$ when the following hold:*

*(i) for $e \in \mathcal{T}_{\mathsf{KA}}$, it holds that $e \equiv^H_{\mathsf{KA}} r(e)$, and*
*(ii) for $e \in \mathcal{T}_{\mathsf{KA}}$, it holds that $[\![e]\!]_{\mathsf{KA}}{\downarrow}^H_{\mathsf{seq}} = [\![r(e)]\!]_{\mathsf{KA}}{\downarrow}^{H'}_{\mathsf{seq}}$.*

*$H$* sequentially reduces *to $H'$ if there exists a sequential reduction from $H$ to $H'$.*

To lift a sequential reduction to a proper reduction, the following class of hypotheses will turn out to be useful.

**Definition 4.33.** *A hypothesis $e \leq f$ with $e, f \in \mathcal{T}_{\mathsf{KA}}$ is called* grounded *if $[\![f]\!] = \{W\}$ for some non-empty word (totally ordered pomset) $W$, and $e \in \mathcal{T}_{\mathsf{KA}}$. We say that a set of hypotheses $H$ is grounded if every $e \leq f \in H$ is grounded.*

*Example 4.34.* Any hypothesis of the form $e \leq \mathsf{a}_1 \cdots \mathsf{a}_n$ for $n > 0$ is grounded. On the other hand, the hypothesis $\mathsf{a} \leq 1$ that we saw in the previous example is not grounded, since the semantics of 1 contains the empty pomset.

The closure of a language of words can be expressed in terms of its sequential closure, provided that the set of hypotheses is grounded.

**Lemma 4.35.** *Let $H$ be grounded. If $L \subseteq \Sigma^*$, then $L{\downarrow}^H = L{\downarrow}^H_{\mathsf{seq}}$. Moreover, for $L, L' \subseteq \mathsf{SP}$, we have that $(L \parallel L'){\downarrow}^H = L{\downarrow}^H \parallel L'{\downarrow}^H$.*

The above then allows us to turn a sequential reduction into a reduction.

**Lemma 4.36.** *Suppose that $H$ sequentially reduces to $H'$. If $H$ and $H'$ are grounded, then $H$ strongly reduces to $H'$.*

## 5 Instantiation to CKA with Observations

In this section, we will present Concurrent Kleene Algebra with Observations (CKAO), an extension of CKA with Boolean assertions that enable the specification of programs with the usual guarded conditionals and loops. We will obtain CKAO as an instance of CKAH by choosing a particular set of hypotheses. First, we define the set of propositional terms or Boolean observations.

**Definition 5.1.** *Fix a finite set $\Omega$ of* primitive observations. *The set of* propositional terms, *written $\mathcal{T}_{\mathsf{BA}}$, is generated by*

$$p, q ::= \bot \mid \top \mid o \in \Omega \mid p \vee q \mid p \wedge q \mid \overline{p}$$

*The relation $\equiv_{\mathsf{BA}}$ is the smallest congruence on $\mathcal{T}_{\mathsf{BA}}$ s.t. for $p, q, r \in \mathcal{T}_{\mathsf{BA}}$, we have*

$$p \vee \bot \equiv_{\mathsf{BA}} p \qquad p \vee q \equiv_{\mathsf{BA}} q \vee p \qquad p \vee \overline{p} \equiv_{\mathsf{BA}} \top \qquad p \vee (q \vee r) \equiv_{\mathsf{BA}} (p \vee q) \vee r$$

$$p \wedge \top \equiv_{\mathsf{BA}} p \qquad p \wedge q \equiv_{\mathsf{BA}} q \wedge p \qquad p \wedge \overline{p} \equiv_{\mathsf{BA}} \bot \qquad p \wedge (q \wedge r) \equiv_{\mathsf{BA}} (p \wedge q) \wedge r$$

$$p \vee (q \wedge r) \equiv_{\mathsf{BA}} (p \vee q) \wedge (p \vee r) \qquad p \wedge (q \vee r) \equiv_{\mathsf{BA}} (p \wedge q) \vee (p \wedge r)$$

*We will write $p \leqq_{\mathsf{BA}} q$ as a shorthand for $p \vee q \equiv_{\mathsf{BA}} q$.*

We write $\mathsf{At}$ for $2^\Omega$, the set of *atoms* of the Boolean algebra. It is well known that every $\alpha \in \mathsf{At}$ corresponds canonically to a Boolean term $\pi_\alpha$, such that every Boolean term $p \in \mathcal{T}_{\mathsf{BA}}$ is equivalent to the disjunction of all $\pi_\alpha$ with $\pi_\alpha \leqq_{\mathsf{BA}} p$ [2]. To simplify notation we identify $\alpha \in \mathsf{At}$ with $\pi_\alpha$.

We can now use $\mathcal{T}_{\mathsf{BA}}$ in defining the terms and axioms of CKAO, which will be given as a CKA over a specific alphabet with the following hypotheses:

**Definition 5.2 (CKAO).** *We define the* terms *of CKAO, denoted $\mathcal{T}_{\mathsf{CKAO}}$, as $\mathcal{T}(\Sigma \cup \mathcal{T}_{\mathsf{BA}})$, that is, as the CKA terms over $\mathcal{T}_{\mathsf{BA}} \cup \Sigma$. We furthermore define the following set of hypotheses over $\mathcal{T}_{\mathsf{CKAO}}$:*

$$\mathsf{bool} = \{p = q : p, q \in \mathcal{T}_{\mathsf{BA}} \text{ s.t. } p \equiv_{\mathsf{BA}} q\} \qquad \mathsf{contr} = \{p \wedge q \leq p \cdot q : p, q \in \mathcal{T}_{\mathsf{BA}}\}$$

$$\mathsf{glue} = \{0 = \bot\} \cup \{p + q = p \vee q : p, q \in \mathcal{T}_{\mathsf{BA}}\} \qquad \mathsf{obs} = \mathsf{bool} \cup \mathsf{contr} \cup \mathsf{exch} \cup \mathsf{glue}$$

*The* semantics *of CKAO is then given by $[\![-]\!]{\downarrow}^{\mathsf{obs}}$.*

The hypotheses $\mathsf{bool}$ contain the boolean identities, and $\mathsf{glue}$ identifies the disjunction with the union (and their respective units as well). $\mathsf{contr}$ specifies that if $p$ and $q$ hold simultaneously, then it is possible to observe them in sequence. Note that the converse inequality is not included: observing $p$ and $q$ in sequence has strictly more behaviour than observing $p$ and $q$ simultaneously, as some intervening action can happen between the two observations.

The above definition gives us the semantics of CKAO as the standard pomset language model obtained from taking the $\mathsf{obs}$-closure of the semantics of CKA. As a matter of fact, we find by Lemma 4.7 that if $e, f \in \mathcal{T}_{\mathsf{CKAO}}$ with $e \equiv^{\mathsf{obs}} f$, then $[\![e]\!]\downarrow^{\mathsf{obs}} = [\![f]\!]\downarrow^{\mathsf{obs}}$; hence, we already have a sound model of CKAO.

To prove completeness, we will use the techniques from the previous section.

*First step: reification.* We start by using reification to rid ourselves of the hypotheses from $\mathsf{bool}$ and $\mathsf{glue}$, and to simplify the hypotheses in $\mathsf{contr}$. To this end, let $\mathsf{contr}'$ be the set of hypotheses given by $\{\alpha \leq \alpha \cdot \alpha : \alpha \in \mathsf{At}\}$. Let $\Gamma = \mathsf{At} \cup \Sigma \subseteq \mathcal{T}_{\mathsf{BA}} \cup \Sigma$. We define $r : \Sigma \cup \mathcal{T}_{\mathsf{BA}} \to \mathcal{T}(\Gamma)$ by setting

$$r(a) = \begin{cases} \sum_{\alpha \leqq_{\mathsf{BA}} p} \alpha & a = p \in \mathcal{T}_{\mathsf{BA}} \\ \mathtt{a} & a = \mathtt{a} \in \Sigma \end{cases}$$

**Lemma 5.3.** *The hypotheses* $\mathsf{obs}$ *reduce to* $\mathsf{exch} \cup \mathsf{contr}'$*.*

*Proof.* By Lemma 4.23, it suffices to show that $r$ is a reification, and that $\mathsf{obs}$ implies $\mathsf{exch} \cup \mathsf{contr}'$. To see that $r$ is a reification, we check the conditions.

(i): If $\mathtt{a} \in \Sigma$, then $r(\mathtt{a}) = \mathtt{a} \equiv^{\mathsf{obs}} \mathtt{a}$ immediately. Otherwise, if $p \in \mathcal{T}_{\mathsf{BA}}$, then we derive $r(p) = \sum_{\alpha \leqq_{\mathsf{BA}} p} \alpha \equiv^{\mathsf{glue}} \bigvee_{\alpha \leqq_{\mathsf{BA}} p} \alpha \equiv^{\mathsf{bool}} p$ and hence $r(p) \equiv^{\mathsf{obs}} p$.

(ii): If $\mathtt{a} \in \Sigma$, then we already know that $r(\mathtt{a}) = \mathtt{a}$. Otherwise, if $\alpha \in \mathsf{At}$, then

$$r(\alpha) = \sum_{\beta \leqq_{\mathsf{BA}} \alpha} \beta = \alpha$$

(iii): This property holds because all hypotheses in $\mathsf{exch} \cup \mathsf{contr}'$ preserve $\Gamma$-languages, i.e., if $e \leq f \in \mathsf{exch} \cup \mathsf{contr}'$ where $[\![f]\!] \subseteq \mathsf{SP}(\Gamma)$, then $[\![e]\!] \subseteq \mathsf{SP}(\Gamma)$ too. It follows that $\mathsf{exch} \cup \mathsf{contr}'$-closure must preserve $\Gamma$-languages.

(iv): We should show that if $e \leq f \in \mathsf{obs}$, then $r(e) \leqq^{\mathsf{exch} \cup \mathsf{contr}'} r(f)$. To this end, we analyse the separate sets of hypotheses that make up $\mathsf{obs}$.

– Let $e \leq f \in \mathsf{exch}$, then $e = (g_{00} \parallel g_{01}) \cdot (g_{10} \parallel g_{11})$ and $f = (g_{00} \cdot g_{10}) \parallel (g_{01} \cdot g_{11})$, for some $g_{00}, g_{01}, g_{10}, g_{11} \in \mathcal{T}$. We then find that

$$r(e) = (r(g_{00}) \parallel r(g_{01})) \cdot (r(g_{10}) \parallel r(g_{11}))$$

$$r(f) = (r(g_{00}) \cdot r(g_{10})) \parallel (r(g_{01}) \cdot r(g_{11}))$$

hence $r(e) \leq r(f) \in \mathsf{exch}$, and therefore $r(e) \leqq^{\mathsf{exch} \cup \mathsf{contr}'} r(f)$.

– Let $e \leq f \in \mathsf{bool}$, then $e = p$ and $f = q$ such that $p \equiv_{\mathsf{BA}} q$. In that case,

$$r(p) = \sum_{\alpha \leqq_{\mathsf{BA}} p} \alpha = \sum_{\alpha \leqq_{\mathsf{BA}} q} \alpha = r(q)$$

– Let $e \leq f \in \mathsf{contr}$; then $e = p \wedge q$ and $f = p \cdot q$ for $p, q \in \mathcal{T}_{\mathsf{BA}}$. Then

$$\begin{aligned} r(p \wedge q) &= \sum_{\alpha \leqq_{\mathsf{BA}} p \wedge q} \alpha \leqq^{\mathsf{contr}'} \sum_{\alpha \leqq_{\mathsf{BA}} p \wedge q} \alpha \cdot \alpha \\ &\leqq \Big( \sum_{\alpha \leqq_{\mathsf{BA}} p} \alpha \Big) \cdot \Big( \sum_{\alpha \leqq_{\mathsf{BA}} q} \alpha \Big) = r(p) \cdot r(q) = r(p \cdot q) \end{aligned}$$

– Let $e \leq f \in \mathsf{glue}$. On the one hand, if $e = p \vee q$ and $f = p + q$, then

$$r(p \vee q) = \sum_{\alpha \leqq_{\mathsf{BA}} p \vee q} \alpha \equiv \sum_{\alpha \leqq_{\mathsf{BA}} p} \alpha + \sum_{\alpha \leqq_{\mathsf{BA}} q} \alpha = r(p) + r(q) = r(p + q)$$

This also establishes the case for $f \leq e \in \mathsf{glue}$. On the other hand, if $e = 0$ and $p = \bot$, then $r(0) = 0 = \sum_{\alpha \leqq_{\mathsf{BA}} \bot} \alpha = r(\bot)$.

To see that $\mathsf{obs}$ implies $\mathsf{exch} \cup \mathsf{contr}'$, it suffices to show that $\mathsf{obs}$ implies $\mathsf{contr}'$. To this end, note that if $e \leq f \in \mathsf{contr}'$, then $e = \alpha$ and $f = \alpha \cdot \alpha$ for some $\alpha \in \mathsf{At}$. We can then derive that $\alpha \equiv^{\mathsf{bool}} \alpha \wedge \alpha \leqq^{\mathsf{contr}} \alpha \cdot \alpha$, and hence $e \leqq^{\mathsf{obs}} f$. □

*Second step: factorising.* Since $\mathsf{contr}'$ satisfies the precondition of Lemma 4.28, we obtain the following.

**Lemma 5.4.** *The hypotheses* $\mathsf{exch} \cup \mathsf{contr}'$ *factorise into* $\mathsf{exch}$ *and* $\mathsf{contr}'$*.*

This means that, by Lemma 4.17 all that remains to do is strongly reduce $\mathsf{exch}$ and $\mathsf{contr}'$ to $\emptyset$; we have already taken care of the former in Theorem 4.26.

*Third step: reducing* $\mathsf{contr}'$*.* In [13], we have already shown that $\mathsf{contr}'$ sequentially reduces to $\emptyset$. Since $\mathsf{contr}'$ is grounded we find the following, by Lemma 4.36.

**Lemma 5.5.** *The hypotheses* $\mathsf{contr}'$ *strongly reduce to* $\emptyset$*.*

*Last step: putting it all together.* Using the above reductions, we can then prove completeness of $\equiv^{\mathsf{obs}}$ w.r.t. $[\![-]\!]{\downarrow}^{\mathsf{obs}}$, and decidability of semantic equivalence, too.

**Theorem 5.6 (Soundness and Completeness of CKAO).** *Let* $e, f \in \mathcal{T}_{\mathsf{CKAO}}$*.*

*(i) We have* $e \equiv^{\mathsf{obs}} f$ *if and only if* $[\![e]\!]{\downarrow}^{\mathsf{obs}} = [\![f]\!]{\downarrow}^{\mathsf{obs}}$*.*
*(ii) It is decidable whether* $[\![e]\!]{\downarrow}^{\mathsf{obs}} = [\![f]\!]{\downarrow}^{\mathsf{obs}}$*.*

*Proof.* For the first claim, we already knew the implication from left to right from Lemma 4.7. Conversely, and for the second claim, first note that that $\mathsf{obs}$ reduces to $\mathsf{exch} \cup \mathsf{contr}'$ by Lemma 5.3. By Lemma 5.4 and Lemma 4.17, the latter reduces to $\emptyset$, if we apply Theorem 4.26 and Lemma 5.5. By Lemma 4.12, we then conclude that $\mathsf{obs}$ is complete and decidable, hence establishing the claim. □

## 6 Discussion

The first contribution of this paper is to extend Kleene algebra with hypotheses [7] with a parallel operator. The resulting framework, concurrent Kleene algebra with hypotheses (CKAH), is interpreted over pomset languages, a standard model of concurrency. We start from simple axioms, known to capture equality of pomset languages [23]. CKAH allows to add custom axioms, the so-called hypotheses. These may be used to include domain-specific information in the language. We develop this framework by providing a systematic way of producing from the hypotheses a sound pomset language model. We also propose techniques that may be used to prove completeness and decidability of the resulting model.

An important instance of this framework is concurrent Kleene algebra (CKA) as presented in [11]. The only additional axiom there, known as the exchange law, may be added as a set of hypotheses. We prove that the resulting semantics coincides with the (subsumption-closed) semantics of CKA and, more interestingly, the completeness proof of [15] can be recovered as an instance of this framework.

The second contribution is a new framework to reason about programs with concurrency: concurrent Kleene algebra with observations (CKAO). CKAO is obtained as an instance of CKAH, where we add the exchange law to model concurrent behaviour, and Boolean assertions to model control flow. The Boolean assertions we consider are as in Kleene algebra with observations (KAO) [13] — in fact, CKAO is a conservative extension of KAO. Using the techniques developed earlier, we obtain a sound and complete semantics for this algebra. While CKAO is similar to concurrent Kleene algebra with tests [12], it avoids the problems of the latter by distinguishing conjunction and sequential composition. CKAO provides the first sound and complete algebraic theory that seems sensible as a framework to reason about concurrent programs with Boolean assertions.

Future work is to explore other meaningful instances of CKAH. Synchronous Kleene algebra [29,26] is a natural candidate for this. We also want to try and design domain specific languages, specifically, a concurrent variant of NetKAT [1,8].

The class of hypotheses considered in this paper for which decidability and completeness may be established systematically is somewhat restrictive; identifying larger classes of tractable hypotheses is a challenging open problem.

Because of the compositional nature of our model, the CKAO semantics of a program contains behaviours that are not possible to obtain in isolation. These behaviours are present to allow the program to interact meaningfully with its environment, i.e., when placed in a context. However, for practical purposes one might want to close the system, and only consider behaviours that are possible in isolation. Studying this semantics remains subject of future work.

In the semantics of concurrent programs with assertions, it would be natural to see atoms as partial instead of total functions. This captures the intuition that a thread might not have access to the complete machine state, but instead holds a partial view of it. Pseudo-complemented distributive lattices (PCDL) have been proposed [12] as an alternative to Boolean algebra, modelling this partiality of information. We leave it to future work to investigate the variant of CKAO obtained by replacing the Boolean algebra of observations with a PCDL.
